# Distributional Validation of Precipitation Data Products with Spatially Varying Mixture Models

**DOI:** 10.1007/s13253-022-00515-0

**Published:** 2022-09-24

**Authors:** Lynsie R. Warr, Matthew J. Heaton, William F. Christensen, Philip A. White, Summer B. Rupper

**Affiliations:** 1 University of California Irvine, Irvine, CA USA; 2 Brigham Young University, Provo, USA; 3 The University of Utah, Salt Lake City, USA

**Keywords:** High Mountain Asia, Latent Variables, Data Augmentation, Ordered Categorical Data

## Abstract

The high mountain regions of Asia contain more glacial ice than anywhere on the planet outside of the polar regions. Because of the large population living in the Indus watershed region who are reliant on melt from these glaciers for fresh water, understanding the factors that affect glacial melt along with the impacts of climate change on the region is important for managing these natural resources. While there are multiple climate data products (e.g., reanalysis and global climate models) available to study the impact of climate change on this region, each product will have a different amount of skill in projecting a given climate variable, such as precipitation. In this research, we develop a spatially varying mixture model to compare the distribution of precipitation in the High Mountain Asia region as produced by climate models with the corresponding distribution from in situ observations from the Asian Precipitation—Highly Resolved Observational Data Integration Towards Evaluation (APHRODITE) data product. Parameter estimation is carried out via a computationally efficient Markov chain Monte Carlo algorithm. Each of the estimated climate distributions from each climate data product is then validated against APHRODITE using a spatially varying Kullback–Leibler divergence measure. Supplementary materials accompanying this paper appear online.

Supplementary materials for this article are available at 10.1007/s13253-022-00515-0.

## Introduction

### Problem Statement and Data

The area known as High Mountain Asia (HMA) is comprised of several important regions, including the Indus, Ganges, and Brahmaputra watersheds. The river systems associated with each of these watersheds provide vital resources for hundreds of millions of people (Lutz et al. 2014; Zhang et al. 2019). Unfortunately, extreme events in these same watersheds also contribute to natural hazards such as flooding and landslides (Immerzeel et al. 2010; Lutz et al. 2014). Hence, added scientific understanding of these watersheds and the over 650 glaciers which feed each watershed is crucial to managing these natural resources and sustaining life in the area.

A principal driver of water availability, glacier mass balance, and glacier runoff in HMA is precipitation. Complicated by the extreme mountainous terrain, in situ observations of precipitation are sparse (Maussion et al. 2014; Palazzi et al. 2013). Hence, the primary scientific understanding of precipitation in HMA comes from digital data products such as climate models and reanalysis data—a data-assimilated combination of observations and climate modeling (Riley et al. 2018; Krishnan et al. 2019). While the value of such digital data products is immeasurable, the fact that these digital products are impacted by an incomplete understanding of the hydrological processes in HMA suggests that they are biased in their characterizations of precipitation in the region (Christensen et al. 2019; Yoon et al. 2019; Mimeau et al. 2019).

**Fig. 1:**
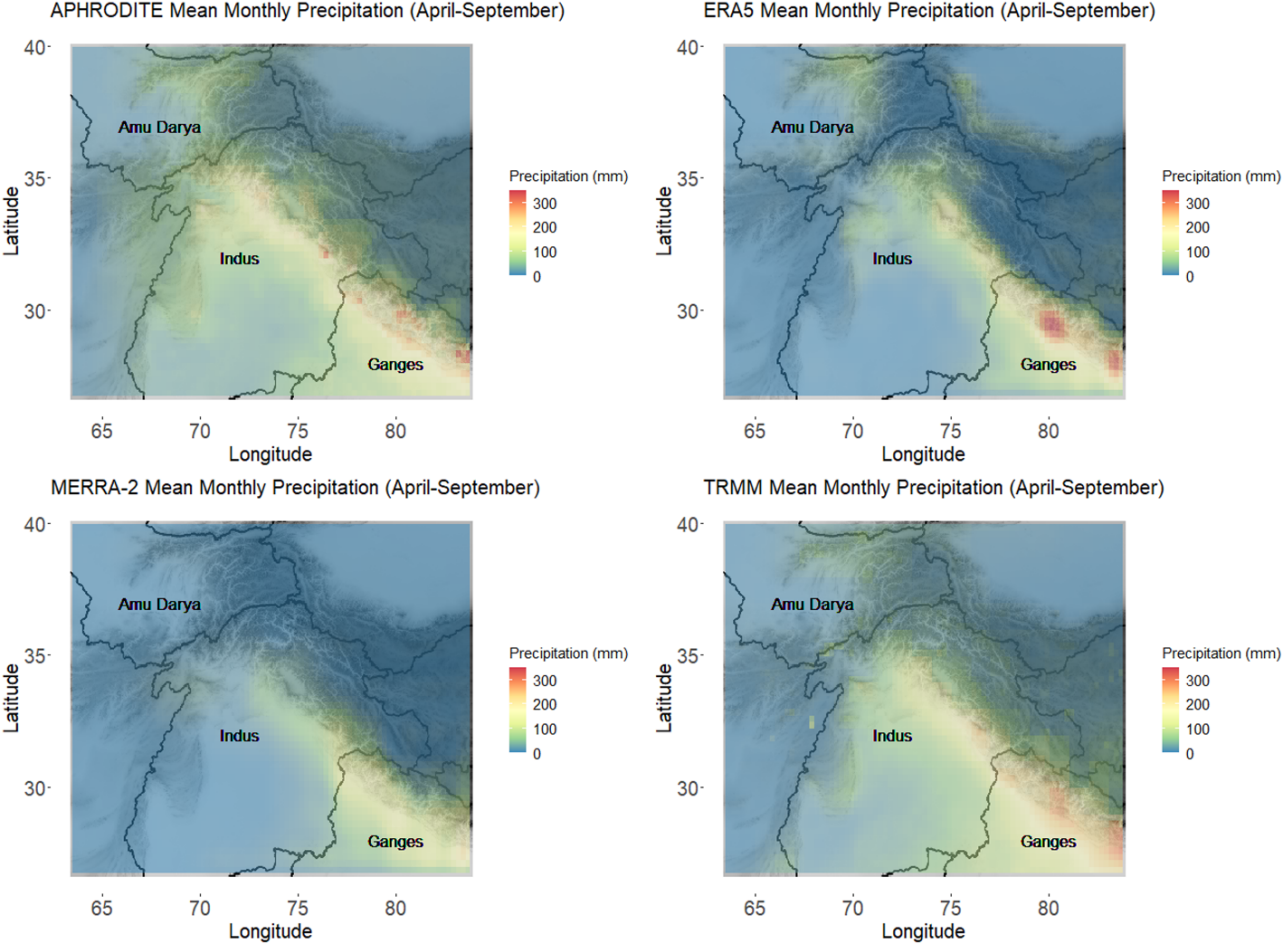
Mean monthly precipitation across the region of interest as estimated by each of the four data products. Watershed boundaries are outlined, and a few are labeled for reference

As an example, consider the following four digital data products that motivate this research. First, the Asian Precipitation—Highly Resolved Observational Data Integration Towards Evaluation (APHRODITE) data product is a continental scale data product based on statistical interpolation of rain gauge data (see https://climatedataguide.ucar.edu/climate-data for more information). Second, the Modern-Era Retrospective analysis for Research and Applications (MERRA-2) data product is reanalysis data based primarily on the assimilation of satellite observations with the GEOS atmospheric forecast model (see Gelaro et al. 2017, for more information). Third, the ERA5 data product is based on data assimilation of a large array of satellite, in situ and snow observations with the ECMWF weather forecast system (see https://www.ecmwf.int/en/forecasts/datasets/reanalysis-datasets/era5 for more information). And, fourth, the Tropical Rainfall Measuring Mission (TRMM) is a purely remote sensing data product (see https://gpm.nasa.gov/missions/trmm for more information). Example data from each product are provided in Figure 1. Note that the products do not all have the same resolution and grid boundaries. For purposes of this research, we regridded ERA5 and MERRA-2 to be on the same grid as APHRODITE and TRMM (with $0.25^{\circ } \times 0.25^{\circ }$ squares) following the methodology of Christensen et al. (2019) in order to have matching, high-resolution grids. A future application of this research would be to explore applying this model to data products with different resolutions.

A careful inspection of Fig. 1, the mean monthly precipitation of the months April through September in the region as estimated by each data product, shows some discrepancies between the data products. For example, there is much disagreement between data products about the precipitation behavior on the Himalayan crest (stretching from the center of the map to the south-east corner). ERA5 indicates some areas with much higher precipitation than the rest of the crest, but these same areas have smaller values in the APHRODITE and TRMM products and don’t show up at all in the MERRA-2 product. Each product also shows varying degrees of precipitation in the areas surrounding the crest. MERRA-2 indicates that large precipitation events are essentially confined to the crest, while the other products, especially APHRODITE, show notable precipitation in various other regions. The products do agree on general trends though, and there are desert regions that all products show as having little to no precipitation.

Discrepancies such as those presented above are prevalent in digital data products. As such, data validation is required prior to using any digital data product for scientific discovery. Data validation is the process of comparing the digital data product to a “baseline” counterpart (typically observational data) to identify any potential strengths and weaknesses of the product and, potentially, correct for any systemic discrepancies. Knowing where, and how, these various data products differ allows scientists to understand where these products might be useful for scientific discovery. Validation also clearly elucidates potential biases that might enter into scientific results by using these products to, for example, inform a climate model.

While a complete review of data validation and bias correction methods for climate models is not possible here (see Maraun 2016; Chen et al. 2019, for holistic reviews), we briefly review the most common approaches to further motivate the contributions of this research. Data validation is most commonly done by comparing summary statistics of the digital data product to the corresponding baseline. For example, linear scaling, or the so-called delta method, validates only discrepancies between the mean and variance of the various data products (see Widmann et al. 2003; Ratna et al. 2017, for examples). However, other data validation approaches include validating quantiles (Teutschbein and Seibert 2012; Jakob Themeßl et al. 2011) or validating correlations amongst variables (Vrac and Friederichs 2015).

### Statistical Challenges

**Fig. 2:**
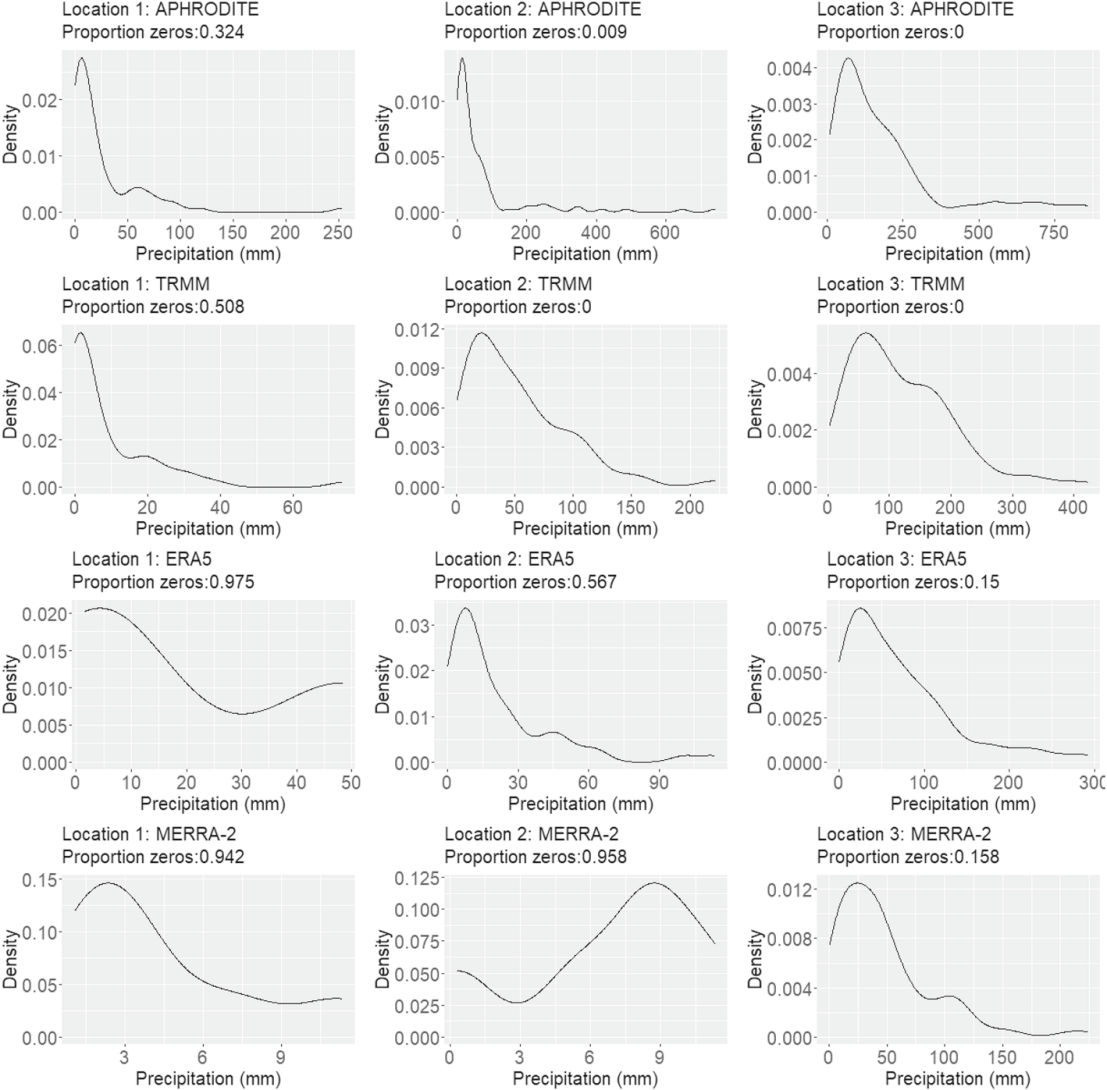
Density plots of mean monthly precipitation for three different locations as represented by each of the data products. (Note that zero values are excluded and summarized by a proportion.) It is clear that precipitation is represented differently by each of the data products and that these differences vary by location. Thus, it is necessary to use a spatially varying model for validation. (See Fig. 3 for a reference of these locations.)

**Fig. 3:**
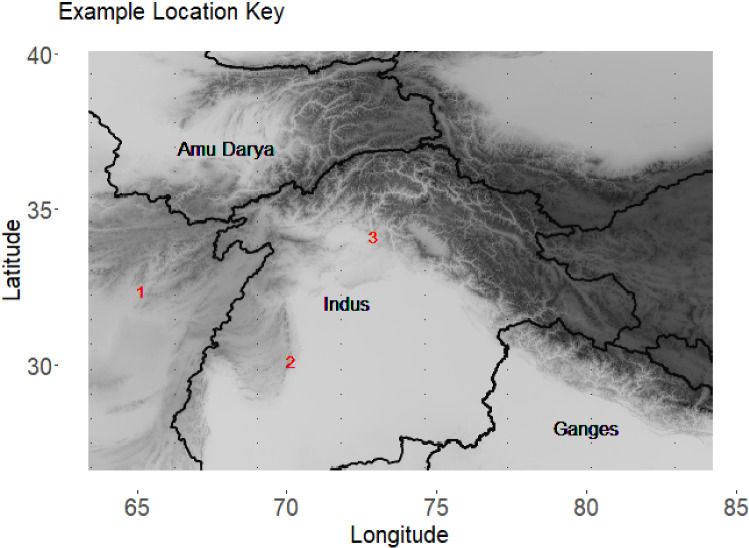
Locations associated with Fig. 2

While data validation is common throughout climate science, the process of data validation presents several interesting statistical challenges that are rarely addressed in the climate literature. First, the distribution of precipitation varies across space. This can easily be seen in Fig. 2 which shows kernel density plots of the four data products at three different locations in the domain. These locations are shown in Fig. 3. The spatial variability seen here also results in correlation between distributions at neighboring locations. The contemporary approaches to data validation mentioned above circumnavigate this problem by performing data validation one location at time. Hence, there is a critical need for validation methods which model a smoothly changing distribution over space to account for spatial relationships.

Second, a single data product may be valid over one subregion of the spatial domain while invalid in others. Using Fig. 1 as an example, the MERRA-2 data product may coincide with APHRODITE in non-mountainous areas while disagreeing with APRHODITE in mountainous regions. With spatially varying discrepancies between products, aggregated metrics (such as an overall mean) mask the true discrepancies between data products. That is, this overall metric would mask any weaknesses (and strengths) in individual data products in capturing local precipitation phenomena such as extreme events. Because of this, it is important to develop methodology that can provide an overall validation measure of the product while maintaining flexibility of validating on smaller spatial domains.

The third challenge is the matter of what to validate a data product on. For example, there are a variety of statistics that can be used for validation such as the mean, median, or quantiles that are appropriate for certain applications (see references given above). However, validating with the mean of the distribution, for example, may be problematic because means are easily influenced by tail behavior such that two similar data products could appear dissimilar due to a few very large precipitation events. While using a median would remedy this issue, such a choice would essentially ignore extremity of tail behavior, which may be scientifically important to consider.

Fourth, the data products considered here contain exact zeros (or are zero-inflated). Exact zeros coupled with positivity of precipitation do not suit the support of any standard distributions. Thus, the complexity of precipitation itself presents various modeling challenges.

### Research Goals and Contributions

In this research, we seek to implement a method for validation of these data products while accounting for the issues discussed above. Specifically, we develop a spatially varying mixture model that allows the distribution of precipitation to vary smoothly over the domain. This is accomplished by allowing the weights of the mixture model to vary smoothly over space. Importantly, by augmenting the parameter space with latent variables, we show that the majority of the parameters in our spatially varying mixture model have conjugate full conditional distributions allowing for ease of computation and good mixing of a Markov chain Monte Carlo (MCMC) sampling scheme.

Using the spatially varying mixture model, we then propose to perform validation using a pointwise Kullback–Leibler (KL) divergence measure. This pointwise KL validation metric can highlight areas where each data product is valid while also allowing aggregation across the spatial domain to produce an overall validation metric for each product. Further, because we model the entire distribution at each spatial location, our proposed methods also have the flexibility of performing validation on any summary of that distribution such as the mean, median or quantile if desired.

The remainder of this paper is outlined as follows: In Sect. 2, the spatially varying mixture model is presented, along with its sampling algorithm and algorithms for calculating validation metrics. In Sect. 3, the spatially varying mixture model fit results are displayed and compared using the various validation metrics. Finally, Sect. 4 contains conclusions and discusses future areas of research.

## Model Description

In this section, we propose our spatially varying mixture model for precipitation along with our chosen metrics to perform data validation. Further, we discuss a latent variable augmentation approach that allows for more convenient posterior sampling.

### Spatially Varying Mixture Model

Let $P_t(s)$ denote the precipitation value at time $t=1 \ldots T$ and location $s \in \mathcal {D}$ for a spatial domain $\mathcal {D} \subset \mathbb {R}^2$. To characterize important features of precipitation such as heavy right skewness and zero inflation, precipitation at each location was modeled as a mixture between a point mass at zero and *K* log-normal distributions. We assume

1 $$\begin{aligned} P_t(s) \overset{ind}{\sim } f(p|s) ={\left\{ \begin{array}{ll} \omega _0(s) &{} \text {if} p=0\\ \sum _{k=1}^K \omega _k(s)\mathcal{LN}\mathcal{}(\mu _k,\sigma _k) &{} \text {if} p > 0 \end{array}\right. } \end{aligned}$$

where $\{\omega _k(s)\}_{k=0}^K$ are spatially varying mixture weights and $\mathcal{LN}\mathcal{}(\mu _k, \sigma _k)$ denotes the log-normal distribution with log-mean $\mu _k$ and log-standard deviation $\sigma _k$. Notably, while any distribution with positive support could be used, the log-normal distribution was specifically selected because of its tendency to have heavy tails, which is especially appropriate for modeling precipitation. For identifiability purposes in mixture models (Celeux 1998; Stephens 2000; Jasra et al. 2005), we order the mixture components such that $\mu _1< \mu _2< \dots < \mu _K$, which will also be important for modeling purposes below.

Notably, the model in Eq. (1) assumes temporal independence and no seasonal variation. Because we subset the data here to the summer season (April–September), directly accounting for seasonal variation is not necessary. Further, given that our data products are monthly precipitation, while not explicitly independent, the temporal dependence present in our data is weak and short-lived (as indicated by an exploratory data analysis). Hence, this assumption is reasonable given our considered data products for this research but may not be appropriate for all data products.

Importantly, the mixture weights in (1) are location-specific, which allows the distribution of precipitation to vary at each location. As such, some locations (e.g., the mountain ridges of HMA) may observe higher amounts of precipitation than others (e.g., the low-lying plains). In modeling the mixture weights, we desire to (i) ensure $\sum _{k=0}^K \omega _k(s) = 1$ for all *s* and (ii) allow $\omega _k(s)$ to vary smoothly over the spatial domain. To accomplish both of these goals, we lean on the fact that the $\{\mu _k\}$ are ordered and follow the approach of Albert and Chib (1993) by defining

2 $$\begin{aligned} \omega _k(s) = \int _{c_{k}}^{c_{k+1}} \frac{1}{\sqrt{2\pi \sigma ^2}}\exp \left( \frac{-(x-\mu _u(s))^2}{2\sigma ^2} \right) dx \end{aligned}$$

where $c_0 = -\infty< c_1 = 0< c_2< \dots < c_{K+1} = \infty$ are a series of cut points and $\mu _u(s)$ is a location-specific mean. Note that, to ensure identifiability, one of the (non-infinite) cutpoints must be fixed; for our purposes, $c_1$ was fixed at 0. Under this parameterization, if $\mu _u(s)$ varies smoothly over space, then $\omega _k(s)$ will also vary smoothly over space. Furthermore, this parameterization allows for all $K+1$ weights $\omega _0(s), \ldots , \omega _K(s)$ to be governed by a single parameter $\mu _u(s)$, greatly reducing the parameter space.

Under (2), spatial smoothing is imposed on the $\{\omega _k(s)\}$ by imposing spatial smoothing on $\{\mu _u(s)\}$. Hence, we parameterize $\mu _u(s)$ using basis function expansions. That is, we let

3 $$\begin{aligned} \mu _u(s)=\mathbf {b}'(s) \varvec{\theta } \end{aligned}$$

where $\varvec{b}(s) = (1, b_1(s),\dots ,b_P(s))'$ is a set of basis functions (defined below) and $\varvec{\theta }$ are the associated coefficients. While any set of spatial basis functions can be used (see Cressie and Johannesson 2008; Banerjee et al. 2008; Nychka et al. 2015; Ma and Kang 2020, for examples), because this research focuses on a gridded data product, we opt to use the Moran basis functions of Hughes and Haran (2013) built from an inverse distance-weighted neighborhood matrix. That is, for the adjacency matrix $\mathbf {A} = \{a_{ij}\}$, we set

4 $$\begin{aligned} a_{ij}&= {\left\{ \begin{array}{ll} 0 &{} \text {if } i=j \\ 1/\Vert s_i - s_j\Vert &{} \text {if } i \ne j \end{array}\right. } \end{aligned}$$

where $\Vert s_i - s_j\Vert$ is the Euclidean distance between each pair of locations. The basis function $\mathbf {b}'(s)$ (with the exception of the intercept term) is then the *s*th row of the eigenvectors of the matrix $(\mathbf {I}-\mathbf {J})\mathbf {A}(\mathbf {I}-\mathbf {J})$ where $\mathbf {J}$ is a matrix of ones. Further, the cumulative sum of the positive eigenvalues of $(\mathbf {I}-\mathbf {J})\mathbf {A}(\mathbf {I}-\mathbf {J})$ can be interpreted to represent the percentage of positive spatial variation explained by the basis functions (eigenvectors). For this research, to balance computational efficiency while still capturing spatial variability, we used $P=94$ basis functions. These 94 basis functions accounted for, approximately, 50% of the theoretical spatial variance, but Hughes and Haran (2013) show that often only 10% of the spatial variation needs to be explained to adequately capture observed spatial patterns.

Under the Bayesian approach, prior assumptions were primarily selected for ease of sampling. The cutpoints $c_2 \ldots c_K$ (which are all of the cutpoints that were not fixed) were transformed in order to sample more easily. These transformed cutpoints (denoted as $\delta _2 \ldots \delta _K$) follow the suggestion by Higgs and Hoeting (2010) and are calculated as:

5 $$\begin{aligned} \delta _k = \text {log}(c_k - c_{k-1}) \text {where} k=1 \ldots K \end{aligned}$$

The transformed cutpoints were assumed to have a uniform prior distribution. The parameter vector $\varvec{\theta }$ was assumed to have a $\mathcal {N}(0,\mathbf {I})$ prior distribution. Note that this prior is somewhat informative. This is intentional: since we impose spatial smoothing on the model through $\mathbf {b}'(s)$ and $\varvec{\theta }$, we enforce some level of spatial smoothing by penalizing values of $\varvec{\theta }$ that are far from 0. Adjusting this prior would be one method of adjusting the strength of the spatial smoothing.

### Latent Variable Augmentation

The model in Sect. 2.1, while flexible, presents some computational challenges when estimating parameters. For example, the $\varvec{\theta }$ parameters would require a Metropolis-type algorithm to sample from the posterior. However, in this section, we propose an equivalent model specification using latent variable augmentation that allows for more convenient posterior sampling for all parameters except for the cut points $c_0, \dots , c_{K}$.

First, let $Z_t(s) \in \{0,\dots , K\}$ represent a latent indicator for the mixture component. That is,

6 $$\begin{aligned} P_t(s) | (Z_t(s) = k) \overset{iid}{\sim } {\left\{ \begin{array}{ll} \delta _0 &{} \text {if} k=0\\ \mathcal{LN}\mathcal{}(\mu _{k},\sigma _{k}) &{} \text {if} k \in \{1,\dots ,K\} \end{array}\right. } \end{aligned}$$

where $\delta _0$ is the Dirac delta function (a point mass) at 0 and $Z_t(s)$ is a discrete random variable with $\mathbb {P}\text {rob}(Z_t(s) = k) = \omega _k(s)$. Notice that marginalizing over $Z_t(s)$ yields Equation (1).

Next, because we label the mixture components based on ordering such that $\mu _1< \mu _2< \cdots < \mu _K$, we can model $Z_t(s)$ as an *ordered* multinomial, spatial random variable. As such, we can employ the methods of Higgs and Hoeting (2010) and Schliep and Hoeting (2015) and further augment the parameter space with another latent variable $U_t(s) \sim \mathcal {N}(\mu _u(s), 1)$ such that,

7 $$\begin{aligned} Z_t(s) = \sum _{k=0}^K k\times \mathbb {1}\{c_{k}< U_t(s) < c_{k+1}\}. \end{aligned}$$

Notably, integrating out the $U_t(s)$, we have $\mathbb {P}\text {rob}(Z_t(s) = k) = \omega _k(s)$ which is equivalently defined by Eq. (2).

Using the above latent variable augmentation, we can now directly sample all model parameters, with the exception of the cut points, from their conjugate complete conditional distributions. The overall Gibbs sampling algorithm is given by Algorithm 1, but here we point out a few important features of the algorithm. First, notice that there is a relationship between the $Z_t(s)$ and $U_t(s)$. That is, given $U_t(s)$, $Z_t(s)$ is known via Eq. (7). Hence, only $U_t(s)$ needs to be sampled but doing so results in a non-conjugate form of the complete conditional distribution. Therefore, Algorithm 1 samples both $Z_t(s)$ and $U_t(s)$ via composition where $U_t(s)$ is integrated out to allow for efficient sampling of $Z_t(s)$. Then, conditional on $Z_t(s)$, the complete conditional distribution of $U_t(s)$ is a truncated Gaussian distribution. 
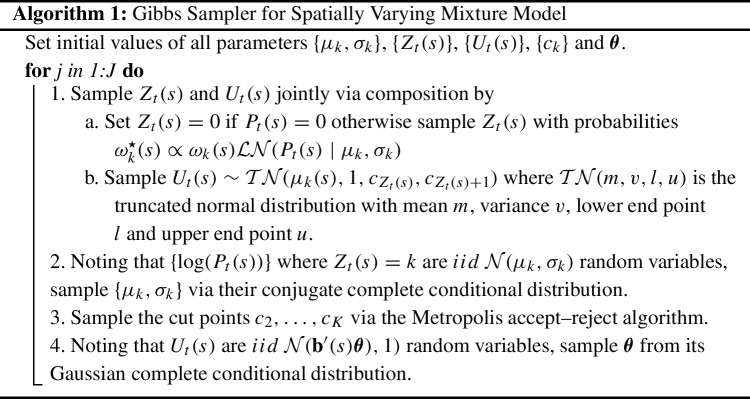


Next, as noted in Albert and Chib (1993), the first cut point $c_1$ needs to be fixed for identifiability reasons (otherwise it is completely confounded with the mean $\mu _u(s)$). Hence, we draw $c_2,\dots ,c_K$ from their complete conditional using an adaptive Metropolis accept–reject algorithm. We again follow the convention of Schliep and Hoeting (2015) and integrate out the latent $U_t(s)$ to sample $c_2, \dots , c_K$ from their posterior distribution given $\{Z_t(s)\}$ and $\mu _u(s)$.

### Validation Metrics

The primary validation metric used here is the Kullback–Leibler (KL) divergence measure (Kullback 1997). This metric, intuitively, measures the amount of information lost when some reference distribution *P*(*x*) is approximated by another distribution *Q*(*x*). Define

8 $$\begin{aligned} D_{KL}(P\vert \vert Q) = \int _{\mathcal {X}} P(x)\text {log}\left( \frac{P(x)}{Q(x)}\right) dx \end{aligned}$$

to be the KL divergence where $\mathcal {X}$ is the support of the distribution (which, in the case of our HMA application, is $[0,\infty )$). First, notice that if $P(x) = Q(x)$ for all *x* then $D_{KL}(P\vert \vert Q) = 0$ suggesting no information loss and a lower bound of zero. However, it is important to note that this metric is asymmetric, meaning that $D_{KL}(P\vert \vert Q) \ne D_{KL}(Q\vert \vert P)$, hence the necessity of choosing a reference distribution to validate against.

For the current research, we consider the KL divergence between the fitted mixture distributions in Eq. (1) for each data product and the model fit to APRHRODITE as the reference distribution. We calculate $D_{KL}$ for each location $s \in \mathcal {D}$ using Monte Carlo integration and the fact that $D_{KL} = \mathbb {E}_P(\log (P(x)) - \log (Q(x)))$. That is, we sample precipitation values $P_t(s) \sim \hat{f}(p\mid s)$ where $\hat{f}(p \mid s)$ is the mixture distribution in Eq. (1) with parameters fixed at their respective posterior means.

We propose that digital data validation using KL divergence is most appropriate because the KL divergence captures all aspects of the distribution of the data. However, there may be specific research questions that are better addressed by comparing certain summary statistics. For example, perhaps the main quantity of interest is the extremes of the distributions in which case we may wish to validate on, say, the 0.95 quantile of the distribution. Because we focus on modeling the entire distribution of the data, we are also able to perform data validation on these other metrics. For example, to validate on a metric other than $D_{KL}$ we can merely (i) sample many $P_t(s)$ from $\hat{f}(p \mid s)$ for each data product, (ii) calculate the chosen summary statistic from each of the two samples and (iii) calculate the difference of the summary statistics of each distribution. Hence, while we focus on $D_{KL}$, our modeling strategy is highly flexible in validating data products on various summary statistics.

## Results

The spatially varying mixture model described in Sect. 2 was fit using Algorithm 1. To assess convergence properties, three chains were run for each data product. For ERA5 and MERRA-2, 150,000 iterations were run with the first 50,000 constituting a burn-in period with the remaining being thinned by 100 to reduce autocorrelation and storage space. In our assessment of convergence, the MCMC algorithm to fit the model to APHRODITE and TRMM took longer to converge. Hence, for those data products 250,000 iterations were run, with a 50,000 burn-in period and thinning every 200th iteration. Code for implementing the sampler, as well as the data used, is available at https://github.com/lynsiewarr/spatiallyvaryingmixture.

In the early stages of this research, the parameters $\{\mu _k, \sigma _k\}$ were estimated as part of the MCMC algorithm outlined in Algorithm 1, but this caused the algorithm to converge extremely slowly while creating issues with identifiability, and it necessitated handling order constraints to maintain consistent labels on the $\mu$ and $\sigma$ parameters. To avoid these issues and prioritize model parsimony, the $\mu$ and $\sigma$ parameters were fixed. By fixing $\{\mu _k, \sigma _k\}$ while allowing the weights $\{\omega _k(\varvec{s})\}$ to be estimated, the mixture components in Eq. (1) are, effectively, a basis function expansion for the underlying distribution. This still results in a model flexible enough to capture the distribution of the data. Fixing $\{\mu _k, \sigma _k\}$ has the added benefit of improved interpretability between data products, as each part of the mixture becomes a similar “precipitation regime.” For example, it is useful to compare how much one data product utilizes the highest precipitation component to how much another utilizes it (which would not be possible if the means of the components were not consistent across data products). To select these values, the nonzero data points from all four data products were divided into *K* equal intervals (rather than percentiles, which would have prevented the components from accurately representing the tails) and the estimators

9 $$\begin{aligned}&\hat{\mu } =\log \left( {\frac{{\text {E}} [X]^{2}}{\sqrt{{\text {Var}} [X]+{\text {E}} [X]^{2}}}}\right) \end{aligned}$$

10 $$\begin{aligned}&\hat{\sigma }^{2}=\log \left( {\frac{{\text {Var}} [X]}{{\text {E}} [X]^{2}}}+1\right) \end{aligned}$$

were used where E[X] was calculated as the middle of the interval, and Var[X] was calculated as the square of half the width of the interval.

**Fig. 4:**
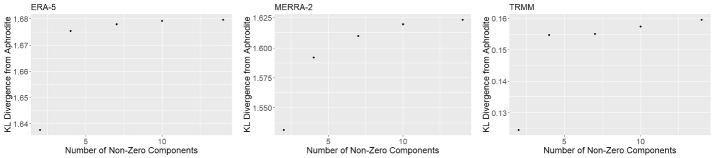
KL divergence from APHRODITE for the different data products for different numbers of components

The total number of nonzero components *K* was selected by examining both DIC and the KL divergence between APHRODITE and each other product, for several different numbers of components. To enable good mixing and avoid identifiability problems, it would be optimal to use as few mixture components as possible while still capturing the differences between products. The DIC values were 1.55e7, 1.39e7, 1.35e7, 1.34e7 and 1.33e7 for $K=2, 4, 7, 10, 14$, respectively. Further, Fig. 4 shows the changing KL divergence for each product across the numbers of components. For ERA5 and TRMM, it appears that most of the differences between the product in question and APHRODITE are captured at $K=4$, as the increase in KL divergence and decrease in DIC slow there. However, we selected $K=7$ (for all data products) to better capture the differences between MERRA-2 and APHRODITE, since the KL divergence for MERRA-2 did not plateau as quickly and the DIC value was lower.

Convergence was assessed by examining trace plots (from one of the chains) and calculating Monte Carlo standard error  (Flegal et al. 2008) and the Gelman–Rubin diagnostic (Cowles and Carlin 1996). While some parameters mixed and converged better than others, overall the convergence diagnostics indicated that we achieved sufficient mixing.

### Model Fit and Qualitative Product Comparison

The first step in performing product validation according to the above methods is ensuring that the fitted mixture distributions match the each data product individually. However, examining the model fit for the spatially varying mixture model is a daunting task since there are distributional fits for each data product across over 4000 locations in our example. Further, in many of the locations, there were very few nonzero precipitation values to assess the model fit on. Thus, to assess model fit, we first examined the difference in the estimated probability of the precipitation being zero ($\omega _0$) compared to the actual proportion of zero values in the data across space (see Figure 1 in the supplementary material). We also examined the estimated distribution compared with a kernel density estimate of the data at a few example locations. Both of these comparisons indicated a model fit that is satisfactory for all the data products. Admittedly, however, both of these comparisons do not necessarily constitute a full model fit evaluation since our model includes spatial smoothing constraints. However, the results from these comparisons give confidence in our data validation below.

**Fig. 5:**
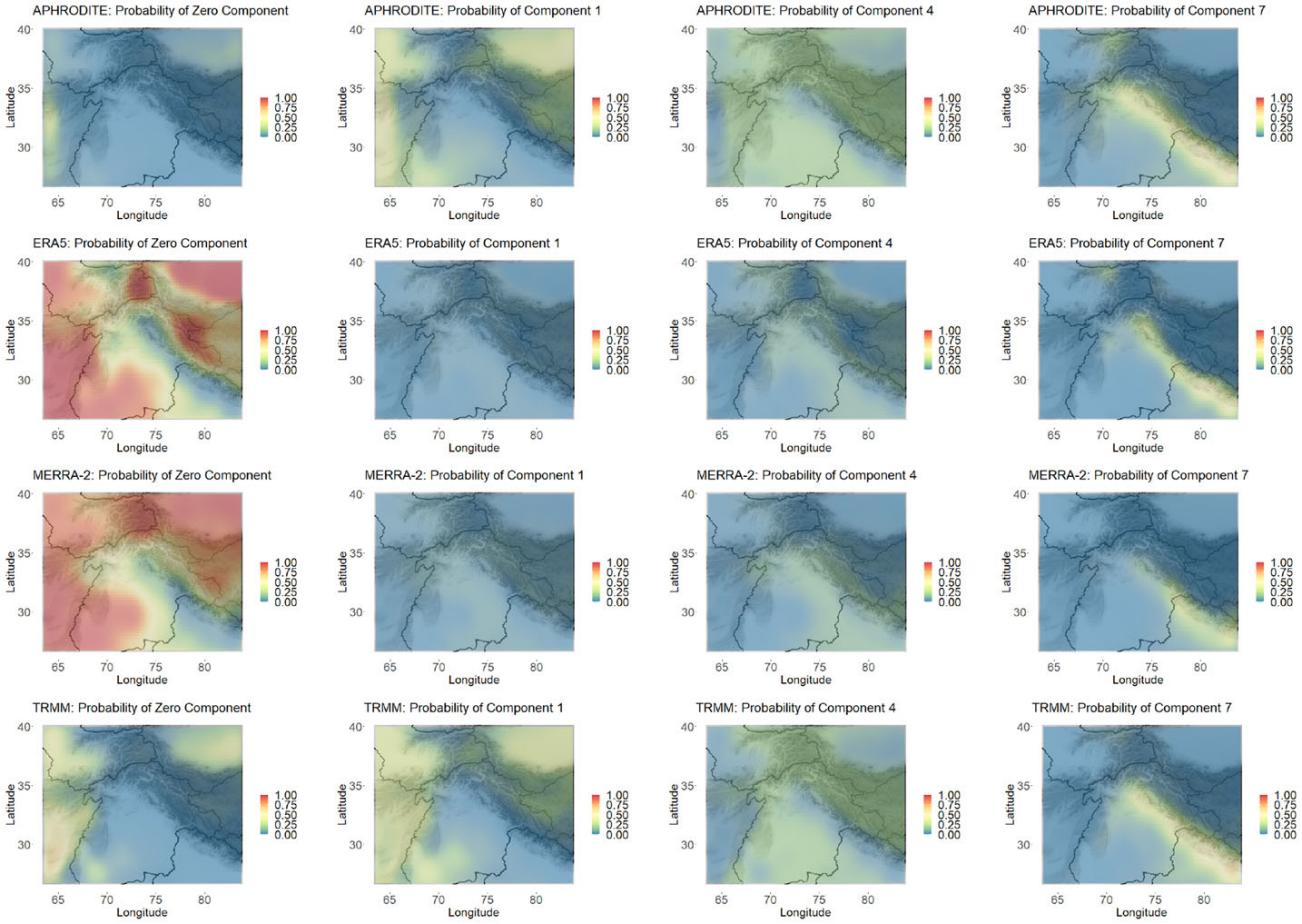
Mixture weights of the zero component and the first, fourth, and last lognormal components of the mixture distribution across space

As a first qualitative validation of the data products, because we fixed the mixture components, we can compare the data products based on the probability of precipitation belonging to any given mixture component. It is clear from Fig. 5 that the model fits for the ERA5 and MERRA-2 data products heavily utilize the zero component in much of the desert regions, while APHRODITE and TRMM use it to a lesser degree. APHRODITE and TRMM appear to detect (or predict) more small, nonzero precipitation events, which are captured in the small and moderate components (components 1 and 4 are shown as examples). The different data product fits all appear to use the most extreme component (component 7) to similar degrees.

**Fig. 6:**
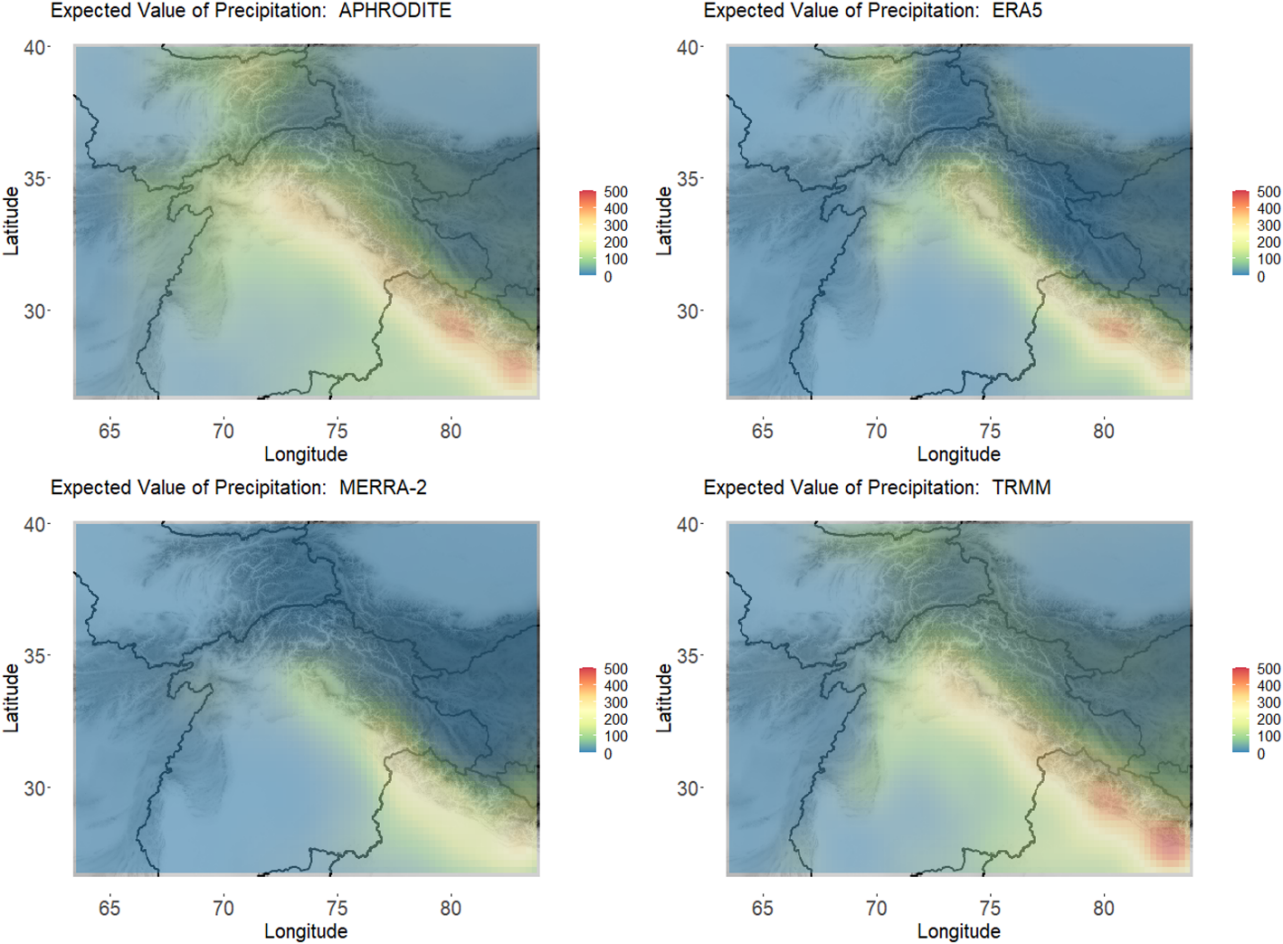
Expected value (mm/month) of the distributions at each location across the region

To further qualitatively compare the different data products, we examined the expected value of the distributions at each location as shown in Fig. 6. These figures also seem to indicate a closer similarity between APHRODITE and TRMM than between APHRODITE and ERA5 or MERRA-2. These differences can be seen in the amount of area that have expected values close to zero, and in the higher extremes along the ridge.

### Quantitative Product Comparison

**Fig. 7:**
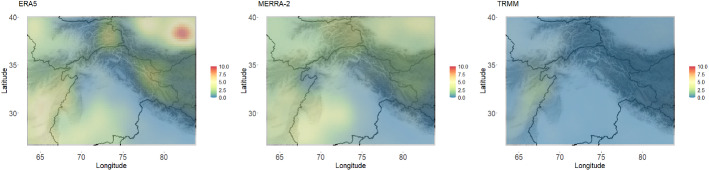
Kullback–Leibler divergence from APHRODITE for each of the three other data products across the region of interest. According to this metric, TRMM has the precipitation distributions closest to APHRODITE’s over most of the region

In order to quantitatively compare ERA5, MERRA-2, and TRMM relative to APHRODITE, the Kullback–Leibler divergence was calculated between the model fit to each of the data products and the model fit to APHRODITE (the reference distribution). The KL divergence was calculated between the distributions at each individual location. The KL divergence across the region for each comparison can be seen in Fig. 7.

Consistent with the qualitative analysis in the previous section, it seems that TRMM is most similar to APHRODITE in nearly every region according to this metric (though parts of the high KL region in TRMM may be outperformed by ERA5). For ERA5 and MERRA-2, the similarities are much closer along the Himalayan crest, while the desert regions are more dissimilar to APHRODITE (likely because of their inability to detect smaller precipitation events as discussed above). As an overall measure of data product validation, the average KL divergence values across the region can be seen in Table 1.

**Fig. 8:**
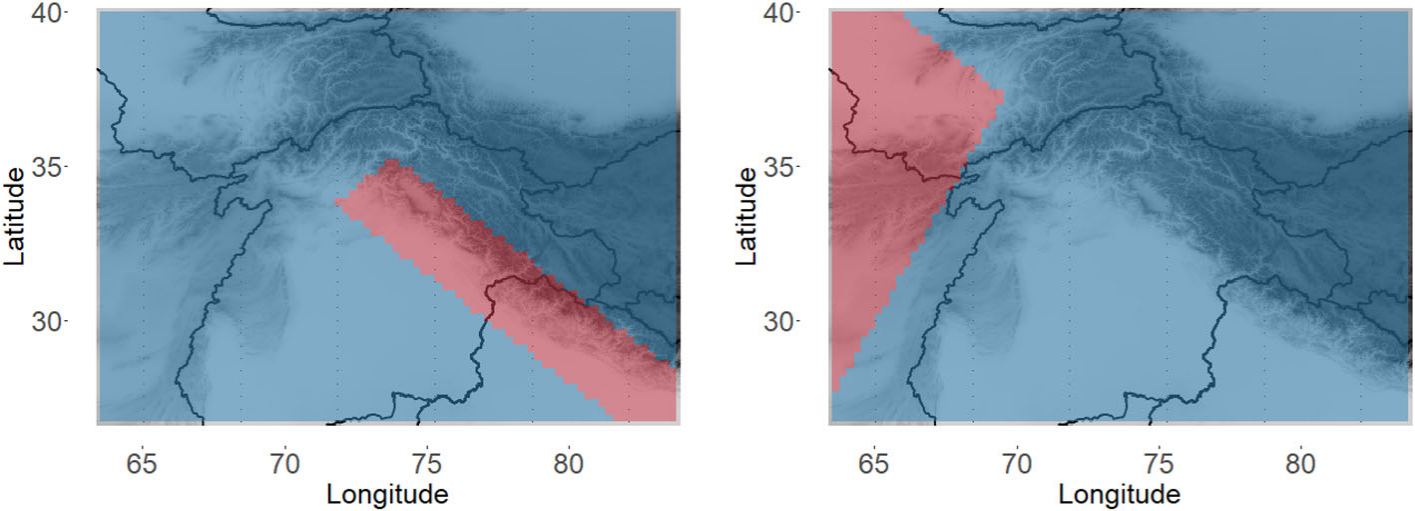
Highlighted regions (red) indicate regions that KL divergence is calculated for in Table 1. Region 1 is on the left, and region 2 is on the right

One particular advantage of our method is that it allows for comparison in certain regions of interest. For example, we can calculate the KL divergence between the model fit for APHRODITE and the model fits for the other data products specifically for the two regions highlighted in Fig. 8. The average KL divergence for each of those regions, as well as the overall KL divergence, is shown in Table 1 for each data product. Notably, each data product seems to perform the best (in terms of comparison to APHRODITE) along the Himalayan crest (Region 1). However, there is more discrepancy between the data products on the western edge of HMA (Region 2).

**Fig. 9:**
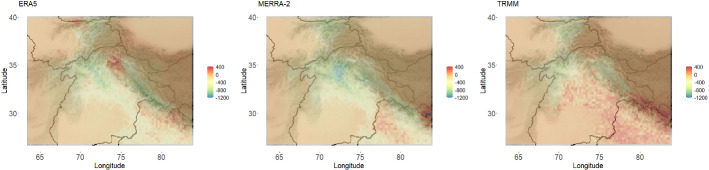
Difference in 95^th^ quantile (mm/month) of precipitation distributions between APHRODITE and the other data products across the region. The difference is calculated as the 95th quantile of the fitted distribution to APHRODITE subtracted from the 95th quantile of the fitted distribution to the other data product

Our validation approach here focuses on estimating the entire distribution of precipitation across the spatial domain. However, because we estimate the distribution, we can easily perform validation of the different data products for various statistics of interest. For example, we can compare the different data products based on the mean, median, or 95th quantile of the fitted distribution. An example of such statistical validation (as opposed to distributional validation) is given in Fig. 9, which displays a spatial map of the difference in the 95th quantile (similar maps showing the difference in mean and median are included in supplementary materials).

**Table 1:** Average KL divergence for the overall region, region 1, and region 2 (as highlighted in Fig. 8) for each of the three data product comparisons to APHRODITE

	Overall	Region 1	Region 2
ERA5	1.678	0.194	2.240
MERRA-2	1.610	0.297	1.979
TRMM	0.155	0.018	0.385

Figure 9 shows that each data product differs quite substantially from APRHODITE in terms of extreme precipitation. That is, along the Himalayan crest, ERA5, MERRA-2 and TRMM all seem to understate extreme precipitation (compared with APHRODITE), while overstating extreme precipitation in the high plains and valleys. It is important to note that the greatest discrepancies in the 95th quantiles occur along the Himalayan crest, while the Himalayan crest has relatively small discrepancies according to the KL divergence metric. This demonstrates the use of KL divergence as a general distribution summary that is not oversensitive to outliers. We would argue this is a better representation of the distribution as a whole.

## Conclusion

In this research, we developed a spatially varying mixture model to estimate the density of precipitation across a heterogeneous region in High Mountain Asia. Through the use of latent variable techniques, we also developed a computationally feasible way of fitting the associated mixture to big data. Having a fitted distribution for precipitation, we then validated various precipitation data products for the region using KL divergence and other distribution summary statistics. Importantly, this validation enables either point-by-point or global comparisons of the products so as to inform scientists on the strengths and weaknesses of each product.

While this work is an interesting first validation of data products, this work does not answer *why* observed differences occur. For example, does elevation explain the difference in the distributions? An interesting follow-up analysis would be to develop some sort of regression model that explains the differences between the distributions. This, in its own right, has various statistical challenges including defining a regression model with a difference between distributions as a response. These are important questions that we hope to address in future research.

Though the various metrics for comparison reveal different information about the distribution similarities between data products, all the validation metrics used in this research indicate that TRMM precipitation is the most similar to APHRODITE precipitation in model fit at most locations. However, the approach used here allows for the identification of specific locations where another data product may be more comparable according to the preferred metric. In other words, using our modeling methods we are able to validate each data product in any given user-defined region.

We specifically recommend using the KL divergence metric for comparison as it evaluates the entire distribution and is not overly sensitive to outliers. Because of these features, we believe it provides a better picture of the water resources available (which is especially important around the Himalayan crest since that is where most of the precipitation occurs).

While the proposed mixture model generally fit the precipitation data well, the $\mu$ and $\sigma$ parameters could also be estimated to potentially improve model fit. In early phases of this research, estimating $\mu$ and $\sigma$ was attempted for this problem but there were issues with convergence and identifiability that made the implementation difficult. Furthermore, in terms of model fit, the locations with small amounts of precipitation were most prevalent because they far outnumber the high precipitation locations. This resulted in poor fits for the high precipitation locations, which may be unacceptable for applications where the right tail is of scientific interest.

For this research, we considered validation of data products on the same resolution. However, different data products are often available on different resolutions and grids. A potential future research avenue is to develop similar methodology that can be applied to different data products at different resolutions and grids.

## Supplementary Information

Below is the link to the electronic supplementary material.

Supplementary file 1 (pdf 1599 KB)
