# Supplementary material for: Distributional Validation of Precipitation Data Products with Spatially Varying Mixture Models
Source: J Agric Biol Environ Stat. 2022 Sep 24;28(1):99–116. doi: 10.1007/s13253-022-00515-0 (PMC9908693; doi:10.1007/s13253-022-00515-0)
Supplement: Supplementary file 1 — (pdf 1599 KB) [file 13253_2022_515_MOESM1_ESM.pdf]

Distributional Validation of Precipitation Data Products with Spatially Varying Mixture Models: Supplementary Materials

2022

Evaluation of Model Fit

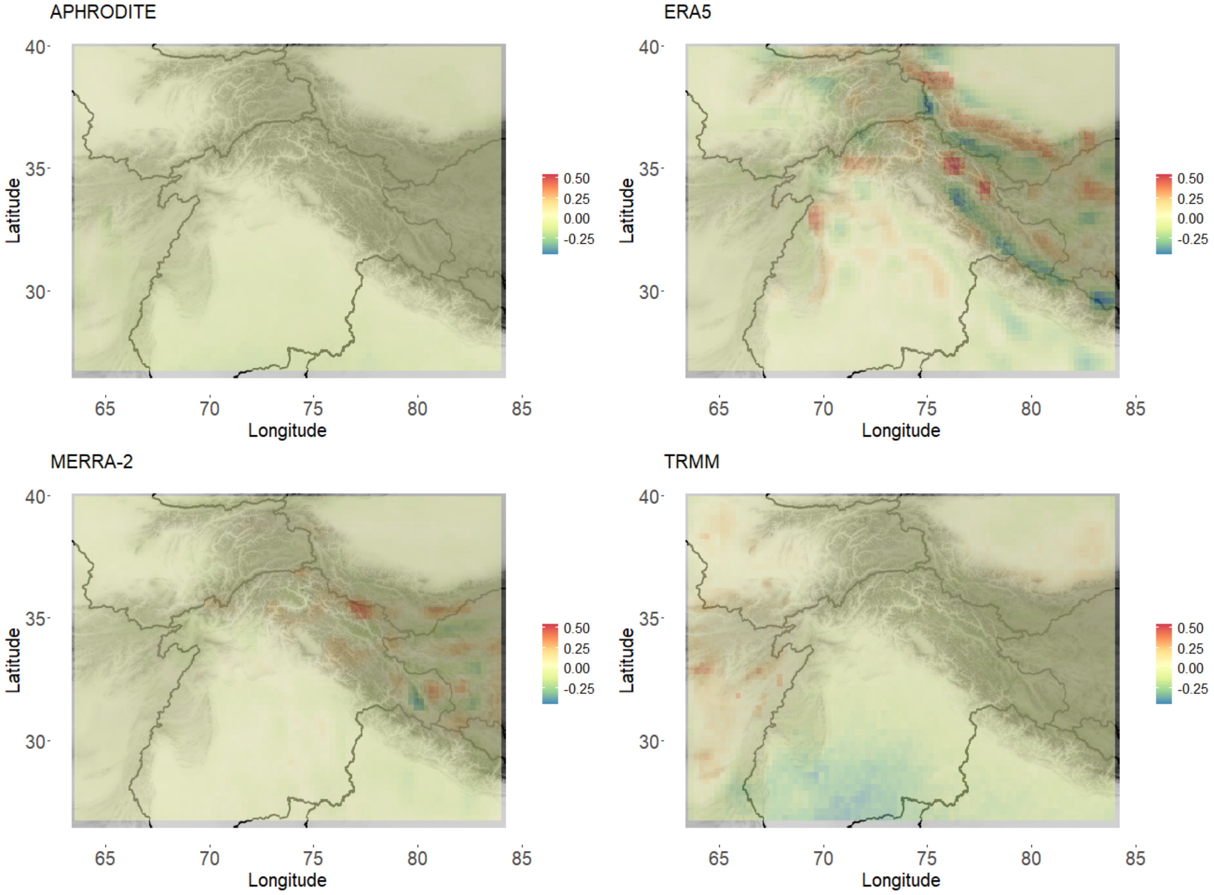

Figure 1: Difference between the estimated probability of precipitation being zero (ω_0) and the actual proportion of zero data at each location. Red locations indicate that our model fits overestimate the proportion of zeros, while blue locations are areas where our model underestimates the propotion of zeros.

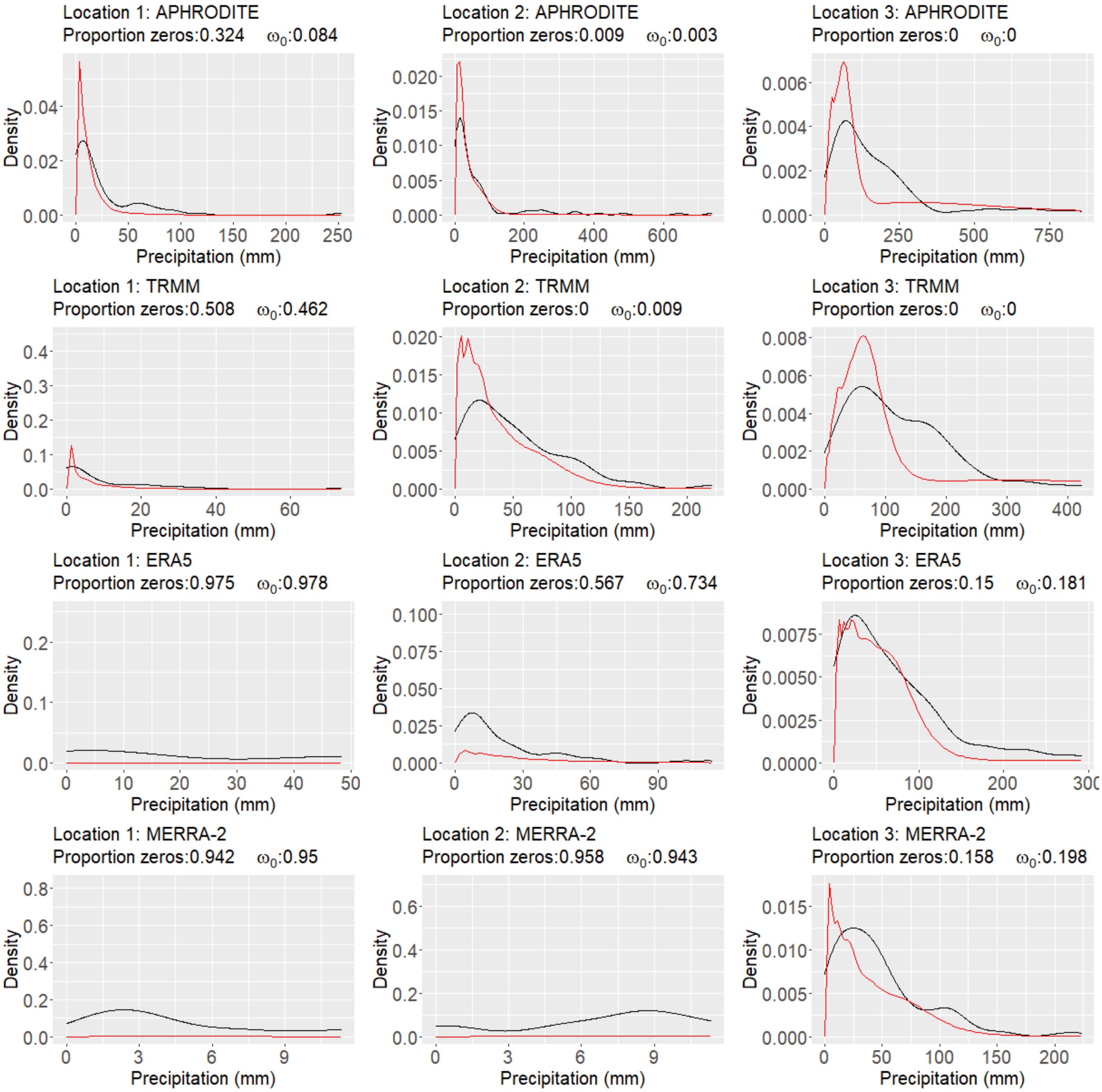

Figure 2: Fitted mixture distributions (red) compared to kernel density estimates of the data at the three example locations. The weights of the zero components are compared to the proportion observed zeroes in the subtitles (zero values are excluded from densities depicted in the plots).

Comparing Across Data Products

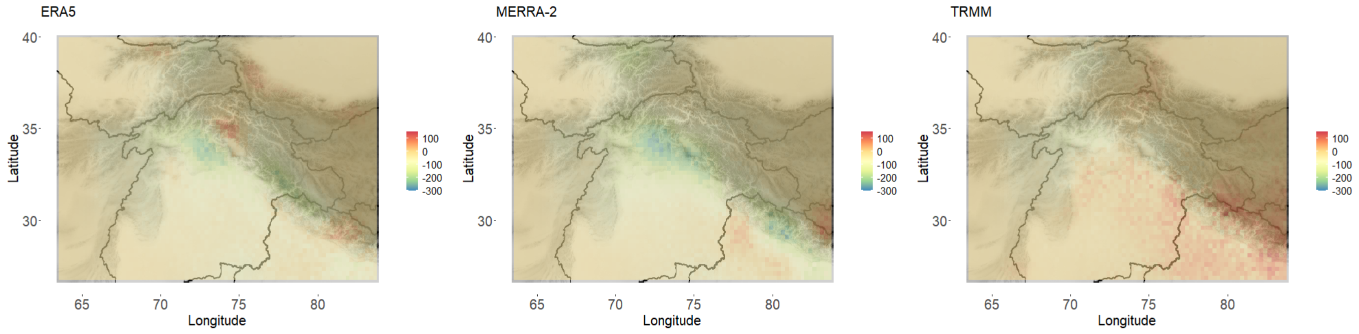

Figure 3: Difference in mean (mm/month) of precipitation distributions between APHRODITE and the other data products across the region. The difference is calculated as the mean of the fitted distribution to APHRODITE subtracted from the mean of the fitted distribution to the other data product. Thus negative values indicate that the data product underestimates the metric compared to APHRODITE in that location.

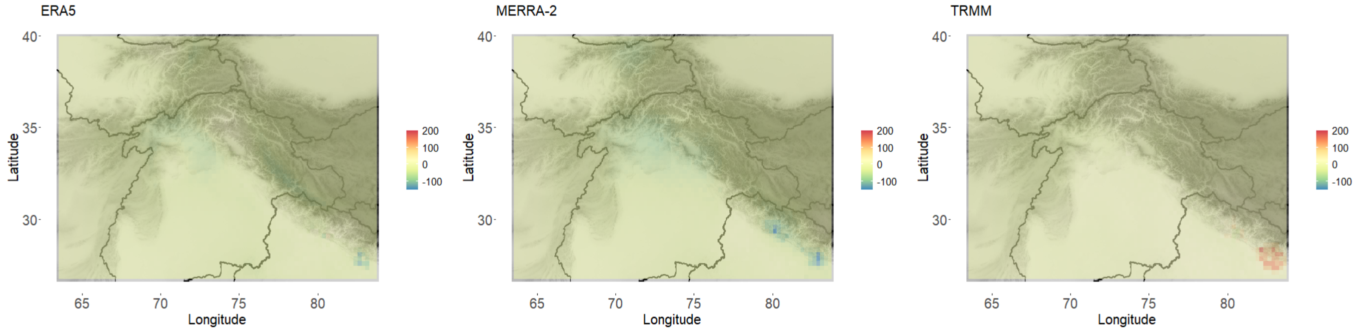

Figure 4: Difference in median (mm/month) of precipitation distributions between APHRODITE and the other data products across the region. The difference is calculated as the median of the fitted distribution to APHRODITE subtracted from the median of the fitted distribution to the other data product.
